# S07-5 Young peoplés perspectives on what makes a sports club health promoting

**DOI:** 10.1093/eurpub/ckac093.038

**Published:** 2022-08-29

**Authors:** Susanna Geidne

**Affiliations:** Sport Department, Örebro University, örebro, Sweden

**Keywords:** Youth, sports clubs, health, participation

## Abstract

**Background:**

In many European countries, about two-thirds of young people participate in sports clubs. However, these numbers peaks in early adolescence and then decrease. In addition, participation in sports clubs looks different in different groups. Participation in sports have the possibility to contribute to young peoplés health in a broad sense, both increasing their physical activity, but also their mental and social health. Sports clubs have also been acknowledged as health promoting settings by researchers and policymakers. Young people participate in sports for different reasons, most common are that it is fun, social, developing and healthy. On the other hand, young people drop out of sports because of coach and teammate relations, but also for organizational reasons like facilities or lack of coaches. A reason can also be too much focus on performance, although this can also be a motivating reason. A major task for sports clubs is to develop sports to maintain the participation of young people. Different perspectives can be used to develop sports clubs activities, among them the novel health-promoting sports clubs approach. Few studies have however explored young people's perspectives on health-promoting sports clubs. The aim of this study is therefore to explore young peoplés perspectives on what makes a sports club health-promoting.

**Methods:**

This cross-sectional study conducted a brief survey in two schools in central Sweden with grade 9 pupils (15-16 years old). The sample consisted of 123 participants (54 % girls, 52 % sport clubs participants, 37 % former sports clubs participants). The questionnaire contained three open-ended questions about what characteristic of a sports clubs makes them feel well, not well and makes them want to stay in sports clubs. The data was analysed with content analysis in combination with statistical analyses.

**Results:**

Early results shows that fun, social dimensions, coaches, but also organizing aspects like amount and ambition of practice are factors that young people think makes a sports club health-promoting.

**Conclusions:**

Health-promoting perspective on sports clubs can from young peoplés perspective include many dimensions and develop sports clubs in this direction could lead to young people staying longer.

